# Intraoperative ligament laxity influences functional outcome 1 year after total knee arthroplasty

**DOI:** 10.1007/s00167-014-3108-0

**Published:** 2014-06-12

**Authors:** Eirik Aunan, Thomas Johan Kibsgård, Lien My Diep, Stephan M. Röhrl

**Affiliations:** 1 Department of Orthopaedic Surgery, Sykehuset Innlandet, Lillehammer, Norway; 2 Orthopaedic Department, Oslo University Hospital, Oslo, Norway; 3 Oslo Centre for Biostatistics and Epidemiology, Oslo University Hospital, Oslo, Norway

**Keywords:** Total knee replacement, Joint instability, Ligament balancing, Monitoring, intraoperative, Knee osteoarthritis, Reference values

## Abstract

**Purpose:**

To find out if there is an association between ligament laxity measured intraoperatively and functional outcome 1 year after total knee arthroplasty (TKA).

**Methods:**

Medial and lateral ligament laxities were measured intraoperatively in extension and in 90° of flexion in 108 patients [122 knees; median age 70 (range 42–83) years]. Mechanical axes were measured preoperatively and at 1-year follow-up. Outcome measures were the Knee Injury and Osteoarthritis Outcome Score (KOOS), the Knee Society Clinical Rating System, the Oxford Knee Score and patient satisfaction. The relationships between laxity and outcome scores were examined by median regression analyses.

**Results:**

Post-operative mechanical axis had a significant effect on the association between ligament laxity and KOOS. Therefore, the material was stratified on post-operative mechanical axis. In perfectly aligned and valgus-aligned TKAs, there was a negative correlation between medial laxity and all subscores in KOOS. The most important regression coefficient (*β*) was recorded for the effect of medial laxity in extension on activities of daily living (ADLs) (*β* = −7.32, *p* < 0.001), sport/recreation (*β* = −6.9, *p* = 0.017) and pain (*β* = −5.9, *p* = 0.006), and for the effect of medial laxity in flexion on ADLs (*β* = −3.11, *p* = 0.023) and sport/recreation (*β* = −4.18, *p* = 0.042).

**Conclusions:**

In order to improve the functional results after TKA, orthopaedic surgeons should monitor ligament laxity and mechanical axis intraoperatively and avoid medial laxity more than 2 mm in extension and 3 mm in flexion in neutral and valgus-aligned knees.

**Level of evidence:**

II.

## Introduction

The effects of ligament laxity on functional outcome after total knee arthroplasty (TKA) are not clearly described in the literature, and defining optimal ligament laxity during TKA is still mostly based on the surgeon’s “feel” and personal experience. Many methods for ligament balancing (soft tissue balancing) have been developed [3, 6, 11, 14, 21, 23, 24, 36, 37], and the current recommendations for ligament balancing are that the gaps should be rectangular and equal. However, it is still not known what the optimal degree of laxity is, and actual intraoperative laxity is typically judged subjectively rather than measured [20, 22].

The deleterious effect of gross instability on prosthetic survival is well documented, and instability is still among the most important reasons for revision knee arthroplasty [27]. The negative effect of overly tight ligaments on knee motion and prosthetic survival has also been described previously [1, 17, 31, 35]. A few studies have reported the influence of ligament balance measured postoperatively on functional outcome after TKA [9, 18, 20]. They concluded that relatively loose knees perform better than tight knees. However, the degree of laxity that leads to subjective instability and poor function is unknown. It is important to bear in mind that instability may also depend on other factors than laxity alone. For example, different adduction moments during walking in varus- and valgus-deformed knees are likely to modify the patient’s perception of laxity.

Most previous studies investigated laxity that was measured clinically or radiographically postoperatively [9, 18, 20, 33]. In order to correct unacceptable results before the end of the surgical procedure, orthopaedic surgeons need information on the relationship between laxity measured intraoperatively and outcome.

Although the literature on the relationship between laxity and functional outcome is non-conclusive, it is likely that such a relationship exists, and if so, it is important for the operating surgeon to have objective data on how and to what degree intraoperative laxity influences outcome. To our knowledge, this is the first study to investigate the relationships between ligament laxity measured intraoperatively, final mechanical axis and functional outcome. The aim of the study was to find out how laxity measured intraoperatively is related to functional outcome 1 year after TKA.

## Materials and methods

Inclusion criteria were patients with primary knee osteoarthritis who were younger than 85 years. Exclusion criteria were patients with severe deformity of the knee, defined as: Bone deformity to such a degree that the bone cuts would damage the ligamentous attachments on the epicondyles; Ligament laxity without a firm end point or to such a degree that ligament releases on the concave side would result in a need for more than 20 mm polyethylene thickness; The combination of bone deformity and ligament laxity resulting in the need for more than 20 mm polyethylene thickness. Excluded were also knees with posterior cruciate deficiency, isolated patella-femoral arthrosis, previous surgery on the knee (except from meniscal surgery and proximal tibial osteotomy) and patients with a severe medical disability preventing them from climbing one level of stairs. Patients not able to fill out the patient-reported outcome measures (KOOS and Oxford knee score) were also excluded.

One hundred and thirty-two patients met the inclusion criteria and twenty-three of these patients were excluded. The reasons for exclusions were as follows (number of patients in parentheses): Severe deformity (1), isolated patella-femoral arthrosis (3), prior surgery on the knee (6), severe medical disability (3), not able to fill out the patient-reported outcome measures (2) and finally, eight patients refused to participate in the study. One 83-year-old woman declined a follow-up visit at 1 year because she was living in a remote area and had experienced no problems with her operated knee. As a result, 122 knees in 108 patients (63 women and 45 men) were investigated. The median age of the patients was 70 (range 42–83) years, and the median body mass index (BMI) of the patients was 29 (range 22–43) kg/m^2^.

All patients underwent surgery consecutively between October 2007 and November 2010 at one community hospital. To ensure conformity in surgical technique, one surgeon (E.A.) was either operating or assisting in every operation.

### Surgical technique

All knees were operated on through a standard midline incision and a medial parapatellar arthrotomy, using a cruciate-retaining prosthesis (NexGen^®^; Zimmer, Warsaw, IN) and a measured resection technique. In order to create a neutral mechanical axis, the valgus angle of the femoral component was set at 5°–8°, depending on the hip–knee–femoral shaft angle, as measured on preoperative standing hip–knee–ankle (HKA) radiographs [10]. Rotation of the femoral component was established by drawing the epicondylar line, the anteroposterior line and the posterior condylar line + 3 degrees external rotation at the distal femoral cut. The average of the three lines was considered to be the true rotational axis. In cases with obvious dysplasia or bony attrition of one or both posterior condyle(s), the posterior condylar line was excluded from the estimation.

Ligament balancing was performed using the technique previously described by Whiteside and colleagues [36, 37]. The aim of the ligament balancing was to achieve medial and lateral condylar lift-off of 1–3 mm in both extension and 90° of flexion.

All operations were performed in a bloodless field with a tourniquet on the proximal part of the thigh.

### Laxity measurements

**Fig. 1:**
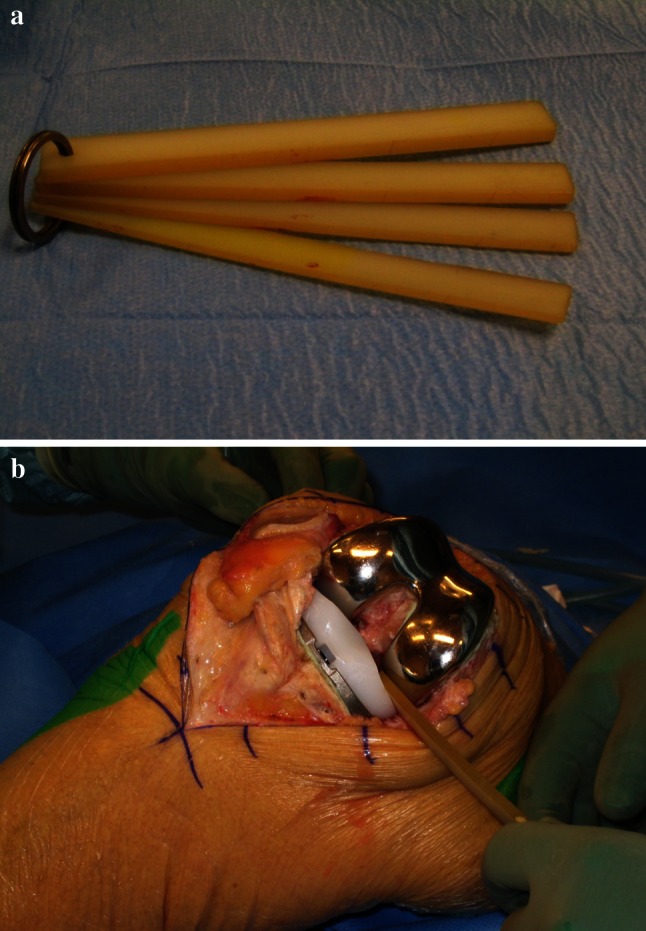
**a** The tool for measuring ligament laxity (condylar lift-off) consists of four spatulas made of polyethylene of increasing thickness [2]. **b** With the knee in 90 degrees of flexion, medial laxity (condylar lift-off) was defined as the distance in the frontal plane from the deepest point of the polyethylene tray to the most posterior point of the femoral condyle. The measurement was performed with the leg in a reversed crossed-leg position under passive valgus stress from the weight of the lower leg with the thickest spatula that could be introduced without force [2]

The method for measuring ligament laxity has previously been described in detail [2]. After implantation of the prosthesis we used a set of four polyethylene spatulas with thicknesses from 2 to 5 mm to measure the medial and lateral laxity (Fig. 1a). With the knee in extension, laxity was defined as the distance in the frontal plane from the deepest point of the polyethylene tray to the most distal point of the femoral condyle. With the knee in 90 degrees of flexion, the same measurements were done between the deepest points of the polyethylene tray to the most posterior point of the femoral condyle. With the knee in extension, the surgeon stressed the ligaments in valgus and varus until a firm end point was felt. Laxity was measured by inserting the thickest spatula possible without using force. If the thinnest spatula could not be inserted and there still was a visible gap, laxity was recorded as 1 mm, in the case of no visible gap, zero was recorded. If laxity was more than 5 mm two or more spatulas were appositioned. In flexion, measurements were performed in the positions described by Tokuhara et al. [34], as follows: Lateral laxity in 90° of flexion was measured in the unilateral cross-legged position under passive varus stress by the weight of the lower leg. Medial laxity in flexion was measured in a similar way with the leg in a reverse cross-legged position (Fig. 1b). All measurements were performed with the patella everted. The reliability (precision) of the measuring method has been tested, and the inter-observer agreement among raters proved to be high with an intraclass correlation coefficient for single measures of 0.88 (95 %confidence interval 0.82–0.92) [2].

### Outcome scores

All patients were clinically evaluated with the Knee Injury and Osteoarthritis Outcome Score (KOOS) [29, 30], the Oxford Knee Score [8] and the Knee Society Clinical Rating System (KSS) [15] preoperatively and at 1-year follow-up. Patient satisfaction was measured on a visual analogue scale (VAS) at 1-year follow-up.

KOOS is a knee-specific, patient-reported outcome measure consisting of 42 questions. It has five separately scored subscales for pain, other symptoms, activities of daily living (ADLs), function in sport and recreation, and knee-related quality of life (QOL). The KOOS has been validated for use in TKR and has been shown to be valid, reliable and responsive [7, 28–30].

The self-administered questionnaires (KOOS, Oxford Knee Score and VAS score) were completed by the patient alone. In bilateral cases (28 knees), the patients were encouraged to consider the knee under investigation when answering the questions.

A physiotherapist, who was blinded to the laxity measures and other details from the operation, assessed the KSS scores including range of motion (ROM).

Mechanical axes were measured preoperatively and at 1-year follow-up on HKA radiographs using the method described by Ewald [10].

The protocol was approved by the Regional Committee on Research Ethics on the University of Oslo (ID number: S-07172d 1.2007.952), and all patients gave their informed consent prior to inclusion in the study.

### Statistical analysis

The mean, standard deviation and range, or median and interquartile range, were given for laxity and outcome scores as appropriate. Numbers and percentages were calculated for categorical variables. The differences between preoperative scores and outcome scores at 1 year were tested with the paired samples *t* test or the Wilcoxon signed-rank test depending on the distribution of paired data.

Initially, the associations between laxity measurements and outcome scores were assessed by Spearman’s rank correlation. Thereafter, confounding variables and effect modifiers known from prior research and biological plausibility were examined statistically using Spearman’s rank correlation. Finally, the relationships between each laxity measurement and the outcome scores were investigated by median regression analysis, adjusting for significant confounders and stratifying on the effect modifier. A median regression model was chosen because of highly skewed data and outliers. The effects of medial and lateral laxity in extension and in flexion on KOOSs are expressed as median regression coefficients. The regression coefficients represent the median changes in outcome scores that can be expected for a 1 mm change in laxity. Two-sided *p* values of <0.05 were considered to be significant. SPSS v.20.0 software (SPSS Inc., Chicago, IL) for Windows was used to carry out descriptive analyses. Median regression analyses were performed with STATA 9.2 statistical software for Windows (StataCorp LP, College Station, TX).

## Results

Alignment and deformity improved from preoperatively to 1 year after surgery (Table 1). Intraoperative ligament laxity measurements showed a tendency towards more laxity in flexion than in extension (Table 2).

**Table 1:** Alignment and deformity measured as deviation from normal mechanical axis in degrees, mean (SD) and range, preoperatively and at 1-year follow-up

	Alignment						*N* (%)
	Varus		Valgus		Neutral		
	Deformity	*n* (%)	Deformity	*n* (%)	Deformity	*n* (%)	
Preoperatively	9.0° (4.8) 1°–22°	98 (80.3)	5.9° (2.7) 2°–13°	20 (16.4)	0	4 (3.3)	122 (100)
At 1 year	2.7° (1.5) 1°–7°	64 (52.5)	2.2° (1.0) 1°–4°	27 (22.1)	0	31 (25.4)	122 (100)

**Table 2:** Ligament laxity (condylar lift-off) measured medially and laterally in extension and in flexion after ligament balancing and implantation of the prosthesis, before closure of the wound, in 122 TKAs. All measurements were performed with the patella everted

	Ligament laxity (mm)	
	Median (IQR)	Range
Extension		
Medially	2 (1–2)	1–5
Laterally	2 (1–3)	0–5
Flexion		
Medially	3 (2–4)	0–9
Laterally	3 (2–4)	0–10

*IQR* inter quartile range

**Fig. 2:**
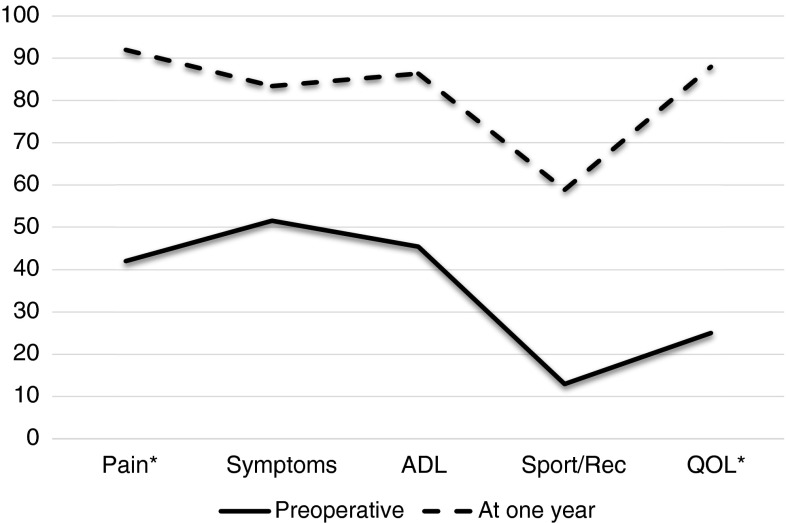
KOOS (including five sub-scores) measured preoperatively and at 1-year follow-up. Mean values are given when *Δ* values (change from preoperative to follow-up at 1 year) where normally distributed, and median values are given when the *Δ* values were skewed. *Δ* values are statistically significant for all subscores (*p* < 0.001). ADL Activities of daily living. Sport/Rec Sport and recreation. QOL knee-related quality of life. * Median values

All function scores improved significantly (*p* < 0.001) at 1 year (Fig. 2; Table 3).

**Table 3:** Knee Society scores, Oxford knee score, knee flexion, knee flexion contracture and patient satisfaction (VAS) measured preoperatively and at 1-year follow-up

	Preoperative	At 1 year	*Δ* (change)	*p*
KSS knee score	34.7 (16.3)	86.2 (13.3)	51.6 (19.0)	<0.001
KSS function score*	67.5 (50.0–80.0)	90.0 (80.0–100.0)	22.5 (10.0 to 36.3)	<0.001**
Oxford knee score^§^	36.90 (7.0)	18.0 (5.8)	−19.0 (8.0)	<0.001
Knee flexion*	120° (110°–128°)	115° (110°–122°)	−5° (−12° to 5°)	0.002**
Knee flexion contracture*	8° (5°–11°)	0° (0°–5°)	−5° (−10° to 0°)	<0.001**
Patient satisfaction (VAS)*		98 (90–100)		

Mean and standard deviation (SD) are given when *Δ* values (change from preoperative to follow-up at 1 year) where normally distributed and as median and interquartile ranges (IQR) when the *Δ* values were skewed. *p* values were tested with paired samples t test if no other indicated

* Skewed data

** Wilcoxon signed-rank test

^§^Oxford score from 12 to 60, the best score is 12

*VAS* visual analogue scale (0–100)

Range of motion (ROM) preoperatively and at 1 year is presented in Table 3. Four knees ended up with less than 90° of flexion and four knees had more than 10° of flexion contracture at the final follow-up.

It was not statistically significant correlation between preoperative mechanical axis or the amount of correction of mechanical axis (from preoperative to postoperative) and outcome measures.

It was no statistical significant correlation between medial and lateral laxity in extension and in flexion and alignment prior or after surgery.

The relationships between laxities and function scores were evaluated in the median regression model: The postoperative mechanical axis proved to interact significantly on the association between medial laxity and outcome for pain (in extension *p* < 0.001 and in flexion *p* < 0.001) and ADL (in extension *p* = 0.008 and in flexion *p* = 0.028) subscores in KOOS. The material was therefore stratified into knees with perfect alignment or valgus alignment (*n* = 58) and knees with varus alignment (*n* = 64) (Table 4). The analyses were adjusted for age, sex and BMI.

**Table 4:** The relationship between ligament laxity (condylar lift-off) and outcome at 1-year follow-up expressed as median regression coefficients with 95 % CI and their *p* values

Outcome (KOOS)	Pain		Symptoms		ADL		Sport/recreation		QOL	
	Coefficient (95 % CI)	p*	Coefficient (95 % CI)	p*	Coefficient (95 % CI)	p*	Coefficient (95 % CI)	p*	Coefficient (95 % CI)	p*
Laxity split by axis										
Medial in extension										
Valgus/neutral *n* = 58	−5.9 (−10.1 to −1.7)	0.006	−2.7 (−8.8 to 3.4)	n.s.	−7.3 (−11.2 to −3.5)	<0.001	−6.9 (−12.5 to −1.3)	0.017	−1.7 (−9.6 to 6.3)	n.s.
Varus *n* = 64	1.2 (−1.1 to 3.4)	n.s.	−0.1 (−6.4 to 6.3)	n.s	2.7 (−0.4 to 5.9)	n.s.	4.2 (−3.8 to 12.0)	n.s.	−0.9 (−7.7 to 5.9)	n.s.
Medial in flexion										
Valgus/neutral *n* = 58	−2.4 (−5.1 to 0.3)	n.s.	−1.9 (−4.3 to 0.4)	n.s	−3.1 (−5.8 to −0.5)	0.023	−4.2 (−8.2 to −0.2)	0.042	−2.2 (−6.0 to 1.6)	n.s.
Varus *n* = 64	0.2 (−1.0 to 1.3)	n.s.	0.4 (−2.4 to 3.2)	n.s	1.0 (−0.9 to 2.8)	n.s.	2.1 (−3.5 to 7.6)	n.s.	−0.8 (−4.2 to 2.5)	n.s.
Lateral in extension										
Valgus/neutral *n* = 58	−1.3 (−5.8 to 3.1)	n.s	−3.0 (−7.4 to 1.4)	n.s	2.2 (−2.3 to 6.7)	n.s.	7.0 (1.0 to 3.0)	0.024	−0.9 (−5.8 to 4.0)	n.s.
Varus *n* = 64	0.1 (−2.7 to 2.9)	n.s.	−5.0 (−9.2 to −0.8)	0.023	0.5 (−4.6 to 5.6)	n.s.	5.6 (−7.6 to 18.8)	n.s.	−3.0 (−10.1 to 4.2)	n.s.
Lateral in flexion										
Valgus/neutral *n* = 58	−0.7 (−3.0 to 1.6)	n.s.	−0.5 (−4.4 to 3.3)	n.s.	−1.8 (−4.8 to 1.2)	n.s.	−2.5 (−6.8 to 1.9)	n.s.	−2.0 (−4.5 to 0.6)	n.s.
Varus *n* = 64	0.1 (−1.4 to 1.5)	n.s	−3.0 (−5.8 to −0.1)	0.041	−1.7 (−5.2 to 1.8)	n.s.	−3.3 (−9.6 to 3.1)	n.s.	−2.7 (−5.9 to 0.4)	n.s.

Ligament laxity was measured intra-operatively medially and laterally in extension and in flexion. Outcome was measured with KOOS (including five subscores). The patients were stratified into two groups depending on the postoperative mechanical axis (alignment) of the operated knee: 58 valgus or neutral knees and 64 varus knees. The analysis was adjusted for age, sex and BMI

* *p* value for the regression coefficient, not corrected for multiple testing. ADL Activities of daily living. QOL knee-related quality of life

In perfectly aligned and valgus-aligned TKAs, there was a negative correlation between medial laxity and all subscores in KOOS (Table 4). The most important regression coefficient (*β*) was recorded for the effect of medial laxity in extension on ADLs (*β* = –7.32, *p* < 0.001), sport/recreation (*β* = –6.9, *p* = 0.017) and pain (*β* = –5.9, *p* = 0.006), and for the effect of medial laxity in flexion on ADLs (*β* = –3.11, *p* = 0.023) and sport/recreation (*β* = –4.18, *p* = 0.042) (Table 4).

In varus-aligned knees, lateral laxity in extension and flexion had a significant negative effect on the symptom subscore in KOOS (*p* = 0.023 in extension and *p* = 0.041 in flexion), but this pattern was not consistent through all subscores (Table 4). The regression coefficients for the KSS and Oxford Knee Score were lower and less consistent than for the KOOSs and did not reach statistical significance.

## Complications

Five perioperative complications occurred. Three were caused by inadvertent saw cuts: one to the popliteal tendon, one to the medial collateral ligament and one to the posterior cruciate ligament. There was one case of atrial fibrillation, and one patient had a small myocardial infarction.

A further two complications were registered within the first year: one patient with lateral knee pain and stiffness underwent neurolysis of the fibular nerve and arthroscopic arthrolysis and mobilization, and one patient with stiffness underwent arthroscopic arthrolysis because of arthrofibrosis, but had poor results and range of motion (8°–78°) at 1 year.

## Discussion

The main finding in this study was that in knees with neutral or slight valgus alignment functional outcome 1 year after TKA was affected negatively by increasing medial laxity in extension and flexion. Additionally, the study shows that postoperative varus/valgus alignment interacts on the association between laxity and functional outcome. This means that the effect of laxity on function depends on the postoperative mechanical axis. It appears that perfectly aligned and valgus-aligned TKAs are more sensitive to increasing medial laxity than varus-aligned TKAs. From a clinical standpoint, it seems reasonable to accept that varus alignment may protect patients with modest degrees of medial laxity from medial instability events, at least in patients with low-grade physical activity. This presumption is supported by gait analysis that has demonstrated that the knee adduction moments are correlated with the mechanical axis of the knee [13]. It is likely that the relatively high adduction moments in varus knees reduce the effect of medial laxity. Vice versa, the low adduction moment in valgus knees may contribute to instability in knees with medial laxity.

Accordingly, one could expect a negative effect of lateral laxity on varus-aligned knees; however, this effect was less pronounced and less consistent through the different subscores (Table 4).

The size of the regression coefficients may be regarded as a measure of the clinical relevance of laxity on function. The minimum perceptible clinical improvement in KOOSs is 8–10 points [30]. Thus, it seems that only a 1–2 mm increase in medial laxity may have a clinically significant impact on subscores in KOOS for ADLs, sport/recreation and pain in patients with perfectly aligned or valgus-aligned knees.

The findings in this study differ from those in earlier reports where functional outcome was found to be better in lax knees. In the studies by Kuster et al. [18] and Edwards et al. [9] laxity measurements were performed in 30° and 20° of flexion, respectively. This might have caused an unknown number of knees with poor function due to too much tightness in extension and/or in 90° of flexion. In a very recent study, Okamoto et al. [26] concluded that the extension gap needs more than 1 mm laxity to avoid postoperative flexion contracture. This finding strengthens the opinion that some laxity is beneficial for the knee function. In our study, we tried to avoid laxity less than one mm and only four out of 488 measurements showed less than one mm laxity.

In the study by Widmer et al. [38] computer navigation was used to assess intraoperative ligament balance. They found a poor association between ligament balance and outcome scores at 1 year. Ligament balance was only assessed with the knee in extension, and in the analysis on the effect of ligament balance on functional outcome, ligament balance was expressed as the change (*Δ* values) in manually tested maximum varus and valgus before and after prosthetic implantation. We consider absolute data on laxity to be more appropriate because the change in ligament balance does not reflect the actual laxity in the knee at the time of functional testing.

Medial–lateral laxity and the mechanical axis were focused on in this study. Subjective stability probably also depends on other factors. Recently, Seah et al. [32] studied the relationship between anteroposterior translation and functional outcome in 100 knees that were replaced with a cruciate-retaining total knee prosthesis. At 2 years of follow-up, patients with a 5–10 mm anteroposterior translation reported significantly better Oxford Knee Scores than patients with less than 5 mm or more than 10 mm anteroposterior translation (*p* = 0.045). Although the loosest knees had the greatest range of motion, they also had the greatest proportion of knees with hyperextension of more than 10°.

In this study, all knees were operated with the measured resection technique and a stepwise ligament-balancing technique where each step increases laxity from roughly zero to 4–5 mm. In order to avoid too tight ligaments or overcorrection (too lax ligaments) some degree of laxity had to be accepted. In contrast, if a pure gap technique is used, laxity can be fine-tuned by further bone cuts. A possible downside of this technique is that these additional bone cuts affect alignment of the knee.

Another important implication of the measured resection technique is that after the mechanical axis has been restored and ligament balancing performed, there should be no correlation between the preoperative degree of deformity and postoperative laxity. This is in concordance with our findings: we found no statistical significant correlation between the preoperative degree of deformity and medial and lateral laxity in extension and in flexion.

The effect of laxity on functional outcome is a major concern in TKA, but it has proved difficult to investigate. There may be various reasons for this. First, the general TKA population is very heterogeneous, with a huge range in age, BMI, physical fitness, activity interests and activity levels. Gender and comorbidities may also be important variables. It is not evident whether all these patients benefit from the same degree of laxity. Second, the choice of outcome measures may be decisive in order to reveal a relationship between laxity and functional outcome after TKA. In this study, the degree of association between laxity and outcome was strongest for the ADL subscore, the sport and recreation subscore and the pain subscore in KOOS. It was not possible to draw firm conclusions based on the KSS score and Oxford Knee Score alone. This may be attributed to a profound ceiling effect in these scores [16], leading to low discriminative capacity.

How tightly should a total knee replacement be balanced? Some authors have proposed guidelines for orthopaedic surgeons to restore normal stability in TKA. Based on a radiographic study measuring knee laxity in 30 healthy, elderly subjects with non-arthritic knees, Heesterbeek et al. [12] recommended varus laxity in flexion between 0° and 7.1° and valgus laxity between 0° and 5.5°. In extension, they suggest that surgeons should aim for varus laxity between 0.2° and 5.4° and valgus laxity between 0.7° and 3.9°.

Bellemans et al. [4] assumed ligament balance to be successful when a 2–4 mm medial–lateral joint line opening was obtained in extension and a 2–6 mm one in flexion.

Our results indicate that medial laxity of more than 2 mm in extension and more than 3 mm in flexion should be avoided. Lateral laxity seems to be more forgiving, especially in knees with neutral or valgus alignment. Varus-aligned knees also seem more forgiving to some minor degree of laxity. Our results also emphasize the importance of having maximal control on the mechanical axis when deciding on the degree of laxity during ligament balancing.

There are several limitations to this study. First, the patient sample was recruited from a general population of TKA patients. Although favourable for the external validity of the study, this also implies that the number and size of confounding factors are high. These confounding factors may disguise possible associations that are not so strong. Second, we observed visible condylar lift-off in almost every measurement. Only four out of 488 measurements recorded no condylar lift-off. When no lift-off is visible, the surgeon does not know how tight the soft tissues are, unless the tension in the ligaments is measured with some kind of mechanical or electronic device. Thus, the results of this study do not apply to knees without visible lift-off when tested for ligament laxity intraoperatively. Third, 14 patients in this study underwent bilateral TKA, and statistical independence between bilateral cases can be questioned. The influence of bilaterality depends on study design and context. In studies comparing outcome after arthroplasty, like in our study, recent papers have concluded that inclusion of bilateral cases does not alter the outcome [5, 25]. Fourth, our method to measure laxity do not distinguish between differences below 1 mm, but in our experience ligament-balancing surgery is not so exact that we feel a need for a more fine-tuned measuring device. The method is based on manual loading of the ligaments in valgus and varus. However, LaPrade and Heikes compared the lateral compartment gapping before and after sequential lateral ligament sectioning on radiographs when varus stress was applied either by a clinician or by a force-application device delivering a 12 Nm moment to the knee [19]. They concluded that both standardized 12-Nm moments and clinician-applied varus stress radiographs provide objective and reproducible measures of lateral compartment gapping.

Fifth, in this study we used CR knees and measured resection technique and our results may not be valid for other types of implants or surgical techniques. Finally, due to the lack of information on the effect size of laxity on functional outcome in former literature sample size calculation was not possible.

The strengths of the present study are its prospective design and the strict consecutive inclusion of patients according to inclusion criteria. Only one patient was lost to follow-up, and no other data are missing. Laxity measurements were performed intraoperatively both in extension and in flexion, enabling the surgeon to correct unacceptable results before finishing the procedure. To the best of our knowledge, this study is the first to describe the effects of ligament laxity, measured directly intraoperatively in millimetres, on functional outcome after TKA.

In a general TKA population, it is likely that many variables will obscure the effect of laxity on outcome, and all patients probably do not benefit from the same degree of laxity. Current outcome scores may not detect instability symptoms adequately. Consequently, further research on the effect of ligament laxity on functional outcome after TKA should focus on more selected patient groups, and both patient-reported outcome measures and performance measures sensitive to instability should be considered.

Until now, the literature has been indecisive on how a TKA should be balanced and surgeons had to depend on their personal experience. This study provides new information enabling orthopaedic surgeons to base their decisions during ligament balancing in TKA on more objective data.

## Conclusion

Final mechanical axis needs consideration during ligament balancing and medial laxity more than 2 mm in extension and 3 mm in flexion must be avoided in neutral and valgus-aligned knees. Varus-aligned knees seem to be more forgiving for medial laxity.
